# Implementation of the asparaginase activity assessment technique for clinical use: experience of a Brazilian Center

**DOI:** 10.1038/s41598-020-78549-y

**Published:** 2020-12-08

**Authors:** Daiane Keller Cecconello, Ciliana Rechenmacher, Isabel Werlang, Priscila Pini Zenatti, Jose Andres Yunes, Ana Paula Alegretti, Claudia Lanvers-Kaminsky, Liane Esteves Daudt, Mariana Bohns Michalowski

**Affiliations:** 1 Graduate Program in Child and Adolescent Health, Universidade Federal do Rio Grande do Sul, Porto Alegre, RS Brazil; 2 Translational Pediatrics Laboratory, Experimental Research Center, Hospital de Clínicas de Porto Alegre, Porto Alegre, RS Brazil; 3 Hospital de Clínicas de Porto Alegre, Porto Alegre, RS Brazil; 4 Centro Infantil Boldrini, Campinas, SP Brazil; 5 University Children’s Hospital of Münster, Münster, Germany

**Keywords:** Cancer, Health care, Oncology, Drug discovery

## Abstract

Acute lymphoid leukemia is a childhood cancer that in high-income countries has event-free survival rates of 80% and global survival rates of 90%. In Brazil these rates are under 70%. This difference may be due to the implementation of supportive care, including the assessment of asparaginase (ASNase) activity. ASNase may cause hypersensitivity reactions and silent drug inactivation. For this reason, ASNase activity monitoring is an essential tool to ensure an effective treatment. Our aim was to implement an ASNase activity measurement technique at a hospital setting. samples from children who were given *Escherichia coli*-derived ASNase were collected. The results of the analyses conducted in our laboratory Hospital de Clínicas de Porto Alegre were compared to those of two institutions: Centro Infantil Boldrini and University of Munster. 262 samples were assessed. The results of the first analyses were compared with those obtained at Centro Infantil Boldrini and showed an ICC of 0.954. Thirty samples were sent to the University of Munster and presented an ICC was 0.960. Our results, when compared to those of national and international centers, showed an excellent agreement. The study was able to implement an ASNase activity test to monitor the treatment.

## Introduction

Acute lymphoid leukemia (ALL) is the most common neoplasm in children. Over the years, improvements in treatment have been observed with the use of multicenter protocols. Whereas high-income countries have event-free survival rates of 80% and global survival rates of 90%, in Brazil these rates are under 70%^1^. This difference is due to several factors, including supportive care with adequate monitoring of the drugs used in the protocols. Asparaginase (ASNase) activity is one of the aspects lacking assessment within our reality in Brazil. Within recent years monitoring increasingly recognized as important to manage ASNase therapy, as it provides a means of identifying patients with sub-ideal activity and provides the information necessary to make adjustments in treatment^2,3^.

ASNase is an enzyme derived from bacteria that has an anti-leukemic function by catalyzing the hydrolysis of the amino acid l-asparagine in l-aspartic acid and ammonia, and is considered a crucial/essential component of therapies for leukemias^4,5^. Three different forms have been developed: one is derived from *Escherichia coli*, another is derived from *Erwinia chrysantemi*, and a third formulation, pegylated asparaginase (PEG ASNase), which is a conjugation of *E. coli* with polyethylene glycol and was created to reduce the immunogenic potential^4,6,7^. Among the adverse effects of ASNase, clinical hypersensitivity reactions were found to occur as a result of the production of anti-ASNase antibodies. These antibodies may also cause rapid enzyme inactivation without clinical signs, referred to as silent inactivation. This phenomenon may generate sub-therapeutic ASNase concentrations leading to a greater chance of relapsed disease^8,9^. Because of these facts, ASNase monitoring is important to predict future allergic reactions or to alert to silent inactivation^1,5,10,11^.

The most sensitive and reproducible method with established clinical use is the assessment of ASNase activity. In addition to being related to the level of asparagine depletion, it has the best correlation with clinical efficacy. For these reasons, it is the method currently indicated for regular use in patient care. Today, regimens that achieve ASNase activity ≥ 0.1 IU/mL are considered to be effective and indicative of a better prognosis^11–14^.

Understanding the reasons for differences in cure rates in ALL patients between Brazil and high-income countries is essential. Several studies postulate that obtaining an effective treatment requires monitoring the activity of medications^6,10,15^. In this study, our aim was to implement an ASNase activity technique with safety, quality, and reproducibility at a hospital setting in Brazil, in order to improve the quality of care for our patients.

## Methods

### Patients and methods

In total, 262 samples were collected from 19 children who were given *E. coli*-derived ASNase according to the Berlin-Frankfurt-Münster BFM 2009 ALL protocol at Hospital de Clínicas de Porto Alegre (HCPA), a university hospital in Porto Alegre, southern Brazil, between April 2017 and December 2017. A minimum 2 mL of blood sample in ethylenediaminetetraacetic acid (EDTA) were collected 24 h and 48 h after each ASNase infusion. Samples were centrifuged at 3670 rpm for 10 min and stored a maximum of 2 h after collection. All procedures and protocols were approved by the Research Ethics Committee of the Hospital de Clinicas de Porto Alegre (CEP/HCPA) under the number CAAE 69093817.4.0000.5327. Furthermore, all experiments were carried out respecting the appropriate guidelines and all individuals who participated in the study confirmed informed consent, including those who were under 18, who had the informed consent of legal guardians.

### Comparative analysis of results

To confirm the successful set up/establishment of the AHA method at our hospital Seventeen analyses were performed at Hospital Boldrini (HB, Campinas, Brazil), which performed the tests only on animals, to practice the technique and establish the critical points of the protocol. Subsequently, 30 samples were sent to the University of Munster (UM, Munster, Germany), a European center of excellence in the analysis of ASNase activity.

**Figure 1:**
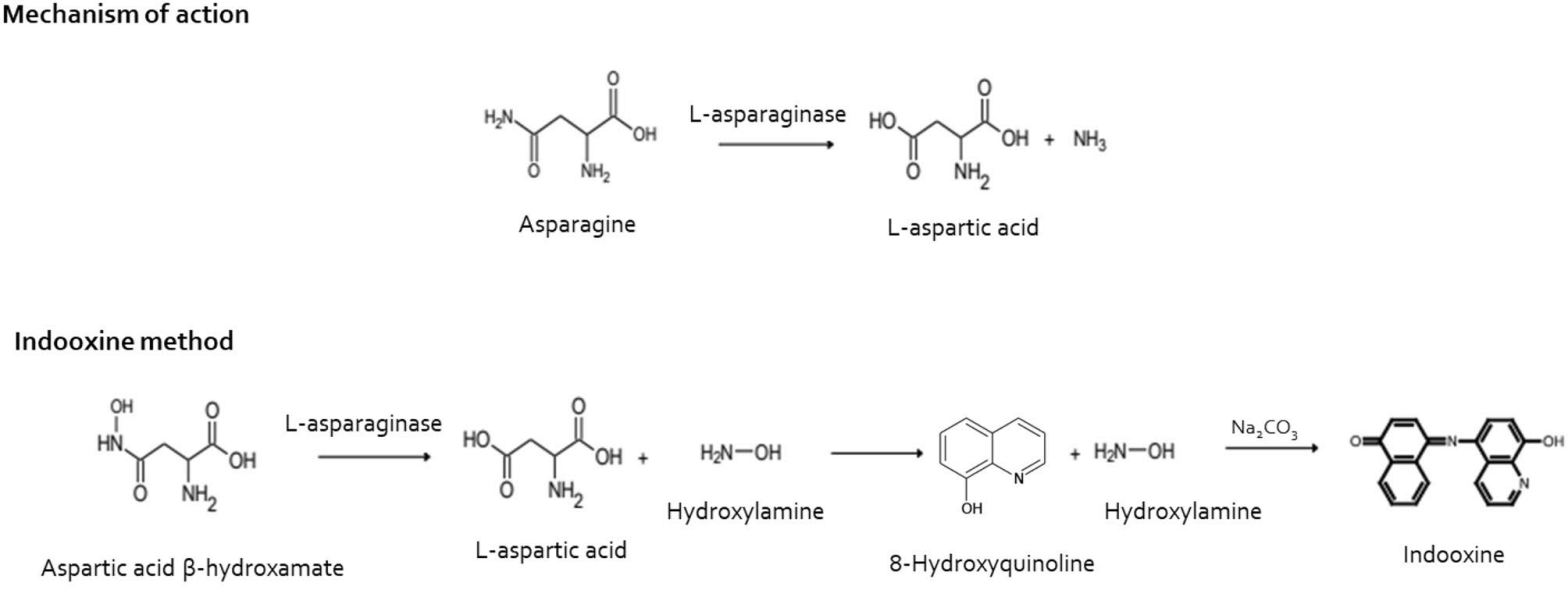
Mechanism of action of asparaginase and reaction of the indooxin method, based on the hydrolysis of l-aspartic b-hydroxamate (AHA), to determine enzyme activity. Figure adapted from Lanvers et al.^10^.

The activity of 262 samples was evaluated in our center, based on the reactions of the below-described method, which were classified according to the activity level as above or below 0.1 IU/mL^16^. The method of analysis used by all centers to determine enzyme activity was based on a technique described by Lanvers et al*.*^10^ which uses aspartic acid B-hydroxamate (AHA) as a substrate for the quantification of ASNase derived from *E. coli*, *Erwinia chrysanthemi*, and PEG ASNase in human plasma. For determination of ASNase activity, we used *E.coli* ASNase concentrations between 0.0025 IU/mL and 0.1 IU/mL for the standard curve. Samples with activity > 0.1 IU/mL were repeated on a standard curve with concentrations between 0.1 and 1 IU/mL of.*E.coli* ASNase. Twenty µl of plasma were diluted with 180 µL of a 2 mM AHA solution dissolved in Tris buffer, pH 7.3 (0.015 M), supplemented with 0.015% (w/v) bovine serum albumin (BSA), and incubated for 30 min at 37 °C. Then, 50 µL of the resulting supernatant were added to a new plate to react with 200 µL of oxin reagent, which consisted of one part of 2% 8-hydroxyquinoline dissolved in absolute ethanol (w/v) and three parts of 1 M sodium carbonate solution. After heating the plate at 95 °C for 1 min and cooling it down for exactly 10 min, absorbance was measured at 690 nm in a SpectraMax M3 equipment (Fig. 1). The standards and samples were analyzed in duplicate, and the control was read in triplicate.

Precision was determined by analyzing plasma samples from the standard curve in concentrations (1; 0.5 and 0.15 IU/mL) with five repetitions (intraday precision = analyzes performed on the same day; inter-day precision = analyzes performed on different days with up to 5 days apart). The acceptance criterion used is the coefficient of variation (CV) < 20% for precision^10^.

## Results

Table 1 shows the results of the 262 samples taken 24 and 48 after the infusion and expresses the results in median and interquartile range. The results of the first 17 analyses were compared to those obtained at HB. The results are shown in Table 2 and had an intraclass correlation coefficient (ICC) of 0.954.

**Table 1:** Medians of enzyme activity evaluated at 24 h and 48 h after infusion.

Time post-infusion (h)	n	Median (IU/mL)	[Q1;Q3] (IU/mL)
24	131	0.066	[0.049; 0.260]
48	131	0.020	[0.011; 0.084]

Data are presented as medians and interquartile ranges.

**Table 2:** Comparative results of *Escherichia coli*-derived ASNase activity (IU/mL) between Centro Infantil Boldrini (CIB, Campinas, Brazil) and Hospital de Clínicas de Porto Alegre (HCPA, Porto Alegre, Brazil).

Sample	CIB IU/mL	HCPA IU/mL
8 A1	− 0.004	− 0.002
17 A1	− 0.004	− 0.002
17 C1	0.025	0.030
17 C2	0.017	0.018
17 C3	0.018	0.022
17 C4	0.016	0.017
17 C5	0.005	0.007
17 C6	− 0.004	− 0.001
17 C7	− 0.004	− 0.002
19 A1	− 0.004	− 0.003
19 C1	0.004	0.006
19 C2	0.011	0.014
19 C3	0.014	0.014
19 C5	0.011	0.011
19 C6	0.012	0.013
19 C7	0.008	0.008
19 C8	− 0.003	− 0.001

*CIB* Centro Infantil Boldrini (Brazil), *IU* International units; Intraclass correlation coefficient = 0.954.

Thirty samples from patients who were given ASNase were then sent to the UM in Germany. The results obtained there are shown in Table 3 and compared to the results obtained at our institution. ICC was 0.960, showing agreement. The precision varied between 5.39 and 5.70% for samples analyzed on the same day (intra-day) and between 5.43 and 6.01% for samples analyzed on different days (inter-days), as shown in Table 4.

**Table 3:** Comparative results of *Escherichia coli*-derived ASNase activity (IU/mL) between the University of Munster (UM, Munster, Germany) and Hospital de Clínicas de Porto Alegre (HCPA, Porto Alegre, Brazil).

Sample	UM IU/mL	HCPA IU/mL
6B5	0.600	0.770
6C5	0.221	0.290
6B6	0.281	0.360
7B1	0.610	0.582
7C1	0.283	0.221
7C2	0.310	0.253
7B3	0.101	0.140
7C5	0.021	0.022
7B7	0.091	0.101
8B2	0.642	0.661
8C2	0.262	0.264
8C3	0.024	0.032
9C8	0.011	0.021
9B9	0.743	0.922
10B2	0.070	0.082
10B8	0.090	0.084
12A1	0.000	0.001
12B2	0.090	0.083
12C5	0.021	0.020
14C3	0.020	0.022
14C5	0.032	0.030
14B9	0.113	0.172
15B1	0.074	0.080
16C1	0.050	0.051
16B3	0.123	0.161
17C2	0.022	0.012
17B5	0.071	0.024
18B2	0.141	0.221
20B1	0.240	0.392

*UM* University of Munster (Germany), *HCPA* Hospital de Clínicas de Porto Alegre (Brazil), *IU* International units; Intraclass correlation coefficient = 0.960.

**Table 4:** Intra-day and inter-day precision for the analytical method.

Range (IU/mL)	Intraday CV (%)	Interday CV (%)
1	5.39	5.43
0.5	5.54	5.56
0.15	5.70	6.01

*CV* coefficient of variation.

## Discussion

ASNase is an important component of the treatment of children with ALL. However, it may cause complications because of some side effects of the enzyme; also, it may be inactivated by the production of antibodies, leading to decreased treatment effectiveness^17^. Adequate levels of ASNase activity are known to result in depletion of asparagine and are of critical importance for patients undergoing treatment for ALL^17,18^. Accurate measurement of serum asparagine in patients may be hindered by continuous enzymatic hydrolysis, since ex vivo metabolism may continue to occur after blood collection, providing an erroneous reading^19,20^.

For this reason, Albersten et al.^21^ recommended monitoring the activity levels of the enzyme ASNase and measuring the levels of antibodies. However, measurement of antibodies is not correlated with clinical repercussion for the treatment of each patient, as there is no clear association between antibody titer and level of enzyme activity^22^. Thus, measurement of ASNase activity levels, as reported in several studies, can be used as a substitute for standardizing asparagine depletion during therapy^12,23^.

To measure the level of substrate conversion, several quantification methods were developed based on the determination of aspartate or ammonia (NH_3_) that was produced. The released NH_3_ can be measured by methods involving reactions with colorimetric reagents, such as Nessler or indophenol, followed by spectrophotometric determination^24^. These methods detect amounts of ASNase in human plasma as low as 0.02 IU/mL^10,25^. The Nessler method exhibits good reproducibility and high level of detection but requires caution because it involves the use of highly toxic reagents. Besides, reaction temperature and color equilibration time affect the color development of the solution, which contributes to variation in the results for this method^24^.

As asparagine depletion is still observed at ASNase concentrations of 0.02 IU/mL, more sensitive methods are needed to determine ASNase activity in human plasma and detect silent inactivation^10^. Methods for determining aspartate include high-performance liquid chromatography (HPLC), electrophoresis assays, and colorimetric assay with hydroxylamine. Lanvers et al*.*^10^ have described the development of an indooxin method for quantification of three different preparations of ASNase in human plasma, a technique that we used in this study. The technique is based on the hydrolysis of AHA, which releases hydroxylamine, which in turn reacts with 8-hydroxyquinoline at alkaline pH. This method produces an intensely green pigment, easily detectable at a range between 690 and 710 nm. This method has a limit of detection as low as 1 × 10^–5^ IU/mL in human plasma but also has a limited working pH range due to hydroxylamine instability above neutrality^10,24^. HPLC-based methods overcome the disadvantages of colorimetric methods, showing excellent reproducibility, precision, and linearity when compared, but they have the disadvantage of resulting in higher costs, making them almost unfeasible for analysis of a large number of samples^24,26^. Therefore, the method described in this study is the most suitable for routine analysis.

The standardization of ASNase quantification by a simple, reliable, fast, and robust method is very useful to overcome the disadvantages of the great disparity between existing methods for determining enzyme activity. With a lack of standardized protocols and pharmaceutical quality control guidelines, standard protocols for measuring the activity of ASNase preparations are rarely reported^23,24^.

ICC is a measure of agreement with ability to identify identical results, being one of the most widely used statistical tools to determine the reliability of measurements. An ICC ≥ 0.75 is considered excellent for data reproducibility^27^. In this study, ICCs of 0.954 and 0.960 ensured agreement between the results of our center and those of HB and UM, respectively. The technique showed reproducibility and precision.

The present study was able to implement an ASNase activity test for monitoring the treatment of patients with ALL, allowing for possible adjustments. Our results, when compared to those of other centers, showed excellent agreement. Importantly, to our knowledge, no laboratory in Brazil had yet routinely monitored the biological activity of this enzyme in patients, although there is already a consensus with expert recommendations for its use.

As far as we know, this study was a pioneer in our country in the assessment of ASNase activity in humans. As previously mentioned, the data were obtained at two different and independent oncology centers in Brazil and at an international center using a well-established protocol. Several reports from European groups have indicated the importance of monitoring ASNase activity to prescribe appropriate treatment, as different methods of administration, formulations, doses, and immune responses may generate substantial variation in ASNase activity levels, as well as the interpersonal response^10,18^. Our study highlights the importance of following the recommendations of international experts for careful handling. The establishment of an appropriate technique allows expanding the availability of this type of assessment to children from other centers.
